# Triggering factors associated with a new episode of recurrent acute anterior uveitis

**DOI:** 10.1038/s41598-021-91701-6

**Published:** 2021-06-09

**Authors:** Nutnicha Neti, Anchisa Pimsri, Sutasinee Boonsopon, Nattaporn Tesavibul, Pitipol Choopong

**Affiliations:** Department of Ophthalmology, Faculty of Medicine Siriraj Hospital, Mahidol University, 2 Wanglang Road, Bangkok Noi, Bangkok, 10700 Thailand

**Keywords:** Risk factors, Uveal diseases

## Abstract

To identify triggering factors for the next inflammatory episode of recurrent acute anterior uveitis (RAAU), a 1:1 case–control study was conducted. We interviewed RAAU patients with recent acute anterior uveitis attack and quiescent controls for their information during a previous month using Srithanya Stress Test (ST-5) and questionnaires about potential triggering factors. Asymptomatic controls were matched for age (± 5 years), sex, and HLA-B27. There were 39 pairs of cases and controls. Patients who recently experienced a uveitis attack demonstrated higher mean ST-5 scores (3.7 ± 2.9 vs 0.7 ± 1.1) and shorter sleep time (6.3 ± 1.4 vs 7.4 ± 0.7 h per day) compared with their controls. In the multivariate conditional logistic regression analysis, ST-5 score ≥ 3 (OR 9.07, 95% CI 1.14–72.16, p = 0.037) and sleep time < 7 h per day (OR 12.12, 95% CI 1.37–107.17, p = 0.025) were more likely to trigger a uveitis attack in RAAU accounted for patients’ age, sex, HLA- B27 positivity, and presence of concurrent anti-inflammatory drugs for co-existing diseases. Other suspected triggering factors were not found to have any significant association. In short, stress and inadequate sleep may lead to the future episode of acute anterior uveitis in RAAU. Both physical and emotional stress management should be advised to RAAU patients to minimize recurrences and further complications.

## Introduction

Uveitis is an intraocular inflammation involving primarily the uveal tissue. This disease is responsible for 10% to 15% of blindness in the United States owing to its consequent ocular morbidities^1^. Anterior uveitis is the most common type of uveitis affecting the anterior segment of the eyes, including iris and ciliary body. According to a previous study, the mean annual incidence and prevalence rates of anterior uveitis were 21 and 69 per 100,000 population, respectively^2^. Of these, immune-mediated recurrent acute anterior uveitis (RAAU) is the most common entity worldwide. The clinical presentations include recurrent acute episodes of ocular pain, eye redness, and photophobia. The degree of inflammation varies from mild anterior chamber reaction to fibrinous hypopyon. Subsequently, repetitive episodes of intraocular inflammation can lead to tissue damage and several serious complications including glaucoma, cystoid macular edema, cataract and eventually permanent visual disability^3^.

Previous studies reported several potential risk factors of RAAU such as HLA-B27^4,5^, structural eye damage^6^, stress^7–10^ and smoking^11^. Whereas the risks for developing RAAU were identified, there was no study clearly stated the predictive factors for the following uveitis episode. Identifying the triggering factors of recurrence will help patients to predict and prevent their upcoming attack. Moreover, understanding the triggers could protect the patients from complications of repeated inflammation.

We, hereby, conducted a matched case–control study to identify the possible triggering factors associated with a recent attack of uveitis in patients with RAAU in comparison with those in quiescence stage of the disease using in person or telephone interview questionnaires regarding stress, sleep time, life events, alcohol consumptions, smoking, exercise time, financial problems and joint pain.

## Methods

### Study population

This study was performed following principles of the international guidelines for human research protections, the Declaration of Helsinki in Good Clinical Practice. Ethical approval was certified by Siriraj Institutional Review Board, Faculty of Medicine, Siriraj Hospital, Mahidol University, Bangkok, Thailand. (SiEC 227/2560). The research project had been registered in Thai Clinical Trials Registry (TCTR20200815001).

In this study, we enrolled patients diagnosed with RAAU who attended the Ophthalmology Service at Siriraj Hospital, Bangkok, Thailand, from August 2017 to July 2018. The inclusion criteria were at least 18 years of age and able to respond to a telephone or face-to-face interview. We excluded patients who had prior eye trauma, accident or eye surgery within six months prior to the interview or had uveitis of any infectious etiology.

The diagnosis of ‘recurrent acute anterior uveitis’ was classified as in the recommendations of the Standardization of Uveitis Nomenclature Working Group^12^. In short, anterior uveitis is an intraocular inflammation involving mainly in the anterior chamber and surrounding ocular tissue which is detectable by a slit lamp biomicroscopic examination. The ‘acute’ course was noted if there was a sudden onset and had a limited duration within 3 months. Patients were diagnosed as having ‘recurrence’ or ‘flare-up’ if they had a second (or additional) episode of active uveitis after three months of no inflammation without treatment. ‘Non-recurrence’ or ‘quiescence’ was described as quiet acute anterior uveitis (no anterior chamber cell) without any recurrences for at least six months without treatment.

### Study design

An age-, sex- and HLA-B27-matched case–control study was conducted with no more than 5-year difference of age. The recently attacked (case) group included RAAU patients who experienced a recent uveitis attack after 3 months or more of quiescence. They were recruited either in person or by phone within a week after being diagnosed by uveitis specialists. We identified potential quiescent (control) participants by searching the age-, sex- and HLA-B27-matched RAAU patients from the hospital’s database. The quiescent RAAU controls were recruited by phone if they did not experience ocular pain, eye redness, photophobia, or diagnosed with uveitis attack within the last 6 months. All participants’ demographic data were obtained from medical records and interview, including age, sex, laterality, HLA-B27 status, underlying diseases, concurrent anti-inflammatory medications, and the number of previous uveitis attack.

The interview questionnaires were performed by one interviewer (AP) either by phone or in-person according to participants’ preference and availability. The questionnaires consist of potential factors associated with a uveitis attack including average sleep time (hours per day), average exercise time (hours per week), smoking, alcohol consumption, prodromal symptoms, low back pain, anxiety, life events, financial problem, eye trauma, accident, history of an illness, and stress. The patients were asked to determine the presence and impact of these factors during one month before the recent attack in the case group or before the interview in the control group.

In this study, we opted the Srithanya Stress Test (ST-5) scoring (Supplement 1) which is a reliable and acceptable tool recommended by the Department of Mental Health, Ministry of Public Health of Thailand for stress assessment in the Thai population^13–17^. The ST-5 evaluated stress levels from sleep problem, loss of concentration, irritability, boredom, and anti-sociality. Each item has the score as 0 (less than once a week), 1 (1–2 times per week), 2 (3–4 times per week), 3 (> 5 times per week). In total, the ST-5 stress score classified level for stress from 0 to 15.

### Statistical analysis

The statistical analyses were performed using PASW Statistics (SPSS) 18.0 (SPSS Inc., Chicago, IL, USA). The unit of analyses was a person. In descriptive statistics, we reported mean with standard deviation (SD) or median with minimal-maximal range (min–max) for continuous data and frequency with percentage for categorical data. In comparing triggering factors between the cases and the controls, we presented their odds ratio (OR) and 95% confidence interval (CI) using univariate conditional logistic regression. A multivariate conditional logistic regression model was performed with the adjustment for the possible triggering factors and pre-determined factors including age, sex, and HLA-B27. A p-value of less than 0.05 was considered statistically significant.

### Human rights statements and informed consent

This study was performed in accordance with the Declaration of Helsinki and approved by the ethical committee on human experimentation of Faculty of Medicine Siriraj Hospital, Thailand. Informed consent was obtained from all participants before included in the study.

## Results

A total of 78 RAAU patients (39 cases and 39 controls) were enrolled in this study and completed the interview questions. Table 1 demonstrated the participants’ demographic data**.** The mean age ± SD was 50.7 ± 11.0 years and 50.9 ± 11.3 years in the case group and the control group, respectively. The gender distribution was predominantly female (64.1%). Most of the participants were HLA-B27 positive (82.1%). There were comparable numbers of previous recurrent uveitis episodes between the two groups (median 2 times, range 2–7 in the case group vs 2 times, range 1–9 in the control group). Most of the participants had unilateral anterior uveitis, 97.4% in cases and 94.9% in controls. Forty-three percent of cases had underlying immunologic diseases, as well as 41.03% in controls. There were slightly higher proportions of patients under concurrent anti-inflammatory medications for coexisting rheumatologic diseases including seronegative spondyloarthropathy and rheumatoid arthritis in the control group (20.5% in controls vs 15.4% in cases).

**Table 1:** Demographic characteristics of recurrent acute anterior uveitis at Siriraj Hospital, Bangkok, Thailand.

Characteristics	Recently attacked RAAU (Case, n = 39)	Quiescent RAAU (Control, n = 39)
Mean age in years (SD)	50.7 (11.0)	50.9 (11.3)
Female (%)	25 (64.1)	25 (64.1)
HLA-B27 positivity (%)	32 (82.1)	32 (82.1)
Unilateral involvement (%)	38 (97.4)	34 (87.2)
Smoking	0	0
Presence of immunological or allergic diseases (%)	17 (43.6)	16 (41.0)
Current anti-inflammatory agents* (%)	6 (15.4)	8 (20.5)
Median number of anterior uveitis episodes (min–max)	2 (2–7)	2 (1–9)
Median A/C cells (min–max)	3 (0.5–4)	0

*RAAU* recurrent acute anterior uveitis, *SD* standard deviation, *HLA-B27* human leukocyte antigen-B27, *A/C* anterior chamber, *min* minimum, *max* maximum.

*Systemic anti-inflammatory agents used in control of coexisting systemic autoimmune diseases.

The recently attacked RAAU group reported statistically significant higher ST-5 scores (3.7 ± 2.9 vs 0.7 ± 1.1) and lower sleeping time (6.3 ± 1.4 vs 7.4 ± 0.7 h per day) than the quiescent group (Table 2).

**Table 2:** Distribution of potential triggering factors for the occurrence of uveitis attack in patients with recurrent acute anterior uveitis (within last 4 weeks before uveitis attack or interview).

Triggering factors	Recently attacked RAAU (Case, n = 39)	Quiescent RAAU (Control, n = 39)
ST-5 score; mean (SD)	3.7 (2.9)	0.7 (1.1)
Sleeping hours per day; mean (SD)	6.3 (1.4)	7.4 (0.7)
Exercise hours per week; mean (SD)	1.5 (1.8)	1.9 (2.1)
Alcohol consumption; n (%)	6 (15.4)	3 (7.7)
Flu-like symptoms; n (%)	4 (10.3)	1 (2.6)
Back or joint pain; n (%)	9 (23.1)	8 (20.5)
Anxiety; n (%)	3 (7.7)	0
Financial problems; n (%)	3 (7.7)	0

*RAAU* recurrent acute anterior uveitis, *ST-5* Srithanya Stress Test, *SD* standard deviation.

Univariate analyses of the possible triggering factors associated with uveitis attack were illustrated in Table 3. Compared to the control group, the ST-5 score ≥ 3 and sleeping time < 7 h per day were statistically significant factors of the present anterior uveitis attack (crude OR 20; 95% CI 2.68–149.02.96; p = 0.003, both). There were no significant differences in exercise time, alcohol consumption, prodromal symptoms, joint pain, anxiety, financial problems, and life events between groups. No participant reported recent smoking, instead most of them are non-smokers (91.4% in cases and 97.1% in controls). Ex-smokers were 8.6% in the recurrent group and 2.9% in the non-recurrent group. No life events such as separation or divorce, occupational change, house moving, death in family and childbirth occurred within one month prior to the uveitis attack in cases or the interview in the controls.

**Table 3:** Univariate conditional logistic regression analyses of triggering factors for the occurrence of uveitis attack in patient with recurrent acute anterior uveitis.

Triggering factors	Crude matched OR	95% CI	p-value
ST-5 score ≥ 3	20	2.68–149.02	0.003
Sleep < 7 h per day	20	2.68–149.02	0.003
Exercise hours per week	0.86	0.65–1.13	0.29
Alcohol consumption	2.5	0.49–12.89	0.273
Flu-like symptoms	–	–	1
Joint pain	1.17	0.39–3.47	0.78
Anxiety	–	–	1
Financial problems	–	–	1

*OR* odds ratio, *CI* confidence interval, *ST-5* Srithanya Stress Test.

Multivariate conditional logistic regression was adjusted for possible triggering factors and age, sex, HLA-B27 typing, and the presence of concurrent anti-inflammatory medications as presented in Table 4. Similar to the results of univariate analysis, both stress (ST-5 score ≥ 3) and inadequate sleep (sleeping time < 7 h per day) were significantly associated with the attack of acute anterior uveitis. Participants with ST-5 stress score ≥ 3 were more likely to have a uveitis attack (adjusted OR 9.07, 95% CI 1.14–72.16, p = 0.037). Also, sleep time less than 7 h per day appeared to be another factor that triggered the uveitis episode (adjusted OR 12.12, 95% CI 1.37–107.17, p = 0.025).

**Table 4:** Multivariable conditional logistic regression model of triggering factors associated with recent acute anterior uveitis attack adjusted for age, sex, HLA-B27 positivity, and concurrent systemic anti-inflammatory medications.

Triggering factors	Adjusted matched OR	95% CI	p-value
ST-5 score ≥ 3	9.07	1.14–72.16	0.037
Sleep < 7 h per day	12.12	1.37–107.17	0.025

*OR* odds ratio, *CI* confidence interval, *ST-5* Srithanya Stress Test.

## Discussion

This study identified that stress and lack of sleep could trigger a uveitis attack in RAAU patients. The odds of uveitis attack within the following month were about nine times in those with stress, and 12 times with sleep deprivation. It appeared that other suspected factors, including exercise time, smoking, joint pain or alcohol consumption, were not significantly correlated with the uveitis attack. However, there was no life event such as childbirth, resettlement, or family member death reported by our participants. We, therefore, could not identify whether a life event could trigger uveitis episode in RAAU.

The National Sleep Foundation recommended 7–9 h of sleep per day for adults aged 26–64 years^18^. A lack of sleep is considered as one of the physical stresses that may alter immune function over time^19–22^. Sleep disturbance and sleep deprivation were claimed to correlate with many autoimmune diseases including rheumatoid arthritis, systemic lupus erythematosus and Behcet’s disease^23^. However, only one study reported the effects of sleep deprivation on ocular inflammation^24^. There was no clear evidence on how lack of sleep activates inflammatory diseases. However, sleep plays a major role in regulating immune functions^25^. Sleep deprivation can lead to increase C-reactive protein level and upregulation of interleukin-6 and tumor necrosis factor^19^. The upsurge of inflammatory cytokines may trigger the inflammatory attack in these vulnerable uveitis patients. In this study, we found that inadequate sleep (less than the recommendation of 7 h per day) showed significant association on triggering an acute uveitis attack in RAAU patients.

On the other hand, the association between psychological stress, immune response and hormones has been widely reported^7,25–31^. According to a meta-analysis, short-term stress can activate the hypothalamic–pituitary–adrenal axis and eventually alter immune response. Stress can induce superoxide surge and suppresses natural killer cell cytotoxicity function which associated with a shifting from T-helper 1 to T-helper 2 response^26^. The correlation between psychological stress and the anterior uveitis attack had been reported in several studies^7,32–35^. A study in the UK included 42 acute anterior uveitis (AAU) patients and 25 controls. The researchers used the General Health Questionnaire (GHQ) to evaluate the stress level. The AAU group had significantly higher GHQ scores than controls (mean 6.8 vs 3.2, p = 0.01)^32^. Another study from the USA recruited 120 AAU patients (80 cases, 40 controls). In contrast, the Cohen 10-item Perceived stress scale (PSS-10) between the groups was not significantly different^36^. In our study, the result revealed that higher stress level (3 or more of the ST-5 score) was correlated with an attack of acute anterior uveitis in this group of Thai population.

From our study, there was an overlapping question between the ST-5 questionnaire and our interview question about sleep. In this regard, the stress score and sleep time might have some collinearity that could interfere the effects of each other. However, they were not identical and could be considered separately. The ST-5 asked about the ‘frequency’ of sleep difficulty or overslept (maximum score of 3) as a representation of emotional instability causing sleep difficulty, while our question asked about the ‘average’ sleep time which was more likely representing physical stress. Additionally, we found their correlation coefficient was very low (rho -0.32, p = 0.005) as well as their variable inflation factor (VIF 1.11). Therefore, sleep time and stress score should not have much effect on each other in the association between them and uveitis attack.

However, in practice, the patients might find it is easier to observe their sleep time than to measure their stress level daily. Therefore, the clinician should inform RAAU patients to have enough sleep to prevent recurrence and be aware of sleeplessness to prepare for the upcoming attack. In addition, self-checking of stress level using ST-5 after noticing lack of sleep could offer further benefit for the patients to predict the attack.

Apart from inadequate sleep and higher stress score, we could not demonstrate the correlation between other physical or psychological events and the new episode of acute anterior uveitis attack. A study by Lin et al. 2010, stated that smoking was strongly associated with all anatomical subtypes of uveitis^37^. In contrast, our data did not find an association between smoking and acute anterior uveitis attack. A plausible explanation might be the limiting number of participants in this study. A larger sample size might better clarify the effect of smoking on recurrences of uveitis. However, due to geographic variation, other variables such as duration of smoking, amount and type of tobacco, ethnicity, might differ largely among each study population. Therefore, it may be challenging to identify the uveitis triggering effect of smoking.

The strength of this study is the use of a parallel control group with age-, sex- and HLA-B27-matched with the cases. Uveitis has different incidence and prevalence based on etiology, gender, HLA-B27 and age^4,38,39^. In order to precisely visualize the unbiased association between risk factors and the acute anterior uveitis attack, we intentionally minimized the bias by using parallel control group as mentioned. Also, both univariate and multivariate statistical analyses were used in this study to identify the potential triggers and their sole effect on uveitis attack. Moreover, to avoid inter-interviewer bias, only one interviewer (AP) was assigned throughout the study.

However, this study contains some limitations, including a recall bias from retrospective questionnaires and small sample size. A large prospective study would help to refine the effect of stress and sleeplessness. In addition, the use of ST-5 for evaluation of stress in our population may not suit the international application; therefore, it limits the generalizability. However, the study indicated the importance of stress evaluation in RAAU, further investigation with international-standard stress scale could improve the generalizability.

## Conclusion

In summary, our study identified stress and inadequate sleep as potential factors triggering uveitis attack in RAAU. These associations emphasize the importance of mental and behavioral management to support the control of anterior uveitis. Being aware of stress level and sleep time may prevent repetitive ocular inflammations and protect the RAAU patients from the potentially sight-threatening complications. Therefore, relieving their stress and having enough sleep time should be added to RAAU patients’ advice.

## Supplementary Information

Supplementary Information.

## Data Availability

All data generated or analyzed during this study are available from the corresponding author on reasonable request.
