# Supplementary material for: Triggering factors associated with a new episode of recurrent acute anterior uveitis
Source: Sci Rep. 2021 Jun 9;11:12156. doi: 10.1038/s41598-021-91701-6 (PMC8190149; doi:10.1038/s41598-021-91701-6)
Supplement: Supplementary file 1 — Supplementary Information. [file 41598_2021_91701_MOESM1_ESM.pdf]

Triggering factors associated with a new episode of recurrent acute anterior uveitis

Nutnicha Neti[1], Anchisa Pimsri[1], Sutasinee Boonsopon[1], Nattaporn Tesavibul[1], Pitipol Choopong[1,*]

	Srithanya Stress Test	0<br>Hardly ever or Never<br>(less than 1 time/week)	1<br>Sometimes<br>(1-2 times/week)	2<br>Usually<br>(3-4 times/week)	3<br>Always<br>(5-7 times/week)
1	How often do you have sleep problems either sleep difficulty or overslept?				
2	How often have you felt that you were unable to concentrate with everyday task?				
3	How often have you felt nervous or irritated?				
4	How often have you felt bored or lack of motivation?				
5	How often have you felt that you had poor social relation or did not want to meet anyone?				

*This version of ST-5 is only translated for publication.

Supplement 1. Srithanya Stress Test (ST-5) in English-translated version*.
